# Constructing the Impostor and Navigating Identity: A Critical Discursive Exploration of the Impostor Phenomenon and Nursing Doctoral Candidates

**DOI:** 10.1111/nin.70144

**Published:** 2026-08-02

**Authors:** Chantel Sando, Amy‐Louise Byrne, Adele Baldwin, Pauline Calleja

**Affiliations:** ^1^ School of Nursing, Midwifery and Social Sciences CQUniversity Rockhampton Queensland Australia; ^2^ College of Medicine and Dentistry James Cook University Cairns Queensland Australia; ^3^ College of Healthcare Sciences James Cook University Townsville Queensland Australia; ^4^ School of Nursing and Midwifery University of Southern Queensland Toowoomba Queensland Australia

**Keywords:** critical discourse analysis, doctoral candidature, gendered discourse, impostor phenomenon, nurse‐academic identity, nursing education, scholarly identity

## Abstract

Impostor phenomenon (IP) is widely recognised among doctoral candidates but remains insufficiently understood, particularly within nursing academia. It is commonly framed as an individual deficit, overlooking the broader social and linguistically mediated experience that shapes scholarly identity. This discursive exploration draws on Fairclough's Critical Discourse Analysis (CDA) to examine micro, meso and macro social orders across diverse textual sources and reflexive accounts, exploring how language and institutional norms position nursing doctoral candidates as impostors. Findings reveal tensions between professional, social and ideological expectations and the formation of a doctoral nurse identity, where dominant discourses individualise systemic constraints, reproduce hierarchies and marginalise nursing expertise through gendered, caring and knowledge‐ownership narratives. Doctoral preparation can disrupt these dynamics by fostering visibility, authority and knowledge production, yet limited support and persistent hierarchies intensify impostor subjectivities. Shifting from deficit framings to critical discourse perspectives enables supervisors, institutions and candidates to cultivate relational, reflective and inclusive practices that mitigate impostor experiences and strengthen nurse‐academic identity. Doctoral education thus becomes a vehicle for structural transformation, advancing nursing's research leadership, policy influence and patient care outcomes.

## 1 Introduction

Nurses comprise the largest professional group in the healthcare workforce, with a scope of practice that extends across models of care, contexts and settings, beyond the provision of bedside care. Nurses contribute to improving health outcomes by applying knowledge gained through robust research, quality education and ongoing professional development (Australian Government 2019; Dobrowolska et al. 2021; Henshaw et al. 2025; Stirling et al. 2024; Walker et al. 2016).

Nurses who undertake doctoral study occupy a complex and contested identity position. As members of the largest professional group in the healthcare workforce (Organisation for Economic Co‐Operation and Development 2025), nurses bring with them deeply embedded professional values grounded in relational practice, care and service (Nursing and Midwifery Board of Australia 2025). Yet doctoral education requires the enactment of scholarly independence, research legitimacy and an authoritative academic voice; expectations that can sit uneasily alongside nursing's historically feminised and practice‐oriented identity (Allan et al. 2008; Clayton‐Hathway et al. 2020; Gill 2020; Hoffmann 1991; Mourão Netto 2023). Within this context, the impostor phenomenon (IP) is frequently reported by nursing doctoral candidates and is often framed as a personal deficit or lack of confidence. Such framings obscure the broader institutional and socio‐professional conditions through which legitimacy, competence and belonging in academia are negotiated, particularly for female‐dominated professions such as nursing (Coelho 2024; Handforth 2022). Professional identity underpins practice and contributes to recruitment and retention, playing an important, albeit often unseen, role in the provision of contemporary, evidence‐based, sustainable healthcare practice. Nursing work extends beyond clinical roles to include education, research and management, all of which require a shift in how nurses perceive themselves and critically analyse the dynamic structural factors shaping professional identity. For example, the transition from clinical practice to academia is not simply a professional progression; it involves a profound reconfiguration of identity. Nurses embarking on higher degrees are expected to demonstrate scholarly independence, research productivity and an authoritative academic voice, which can conflict with the relationally oriented practice and human‐centred skills central to nursing (Baldwin 2016; Dobrowolska et al. 2021; Lamichhane 2025; McAlpine et al. 2014; Stirling et al. 2024). The formation of a scholarly identity is thus a complex negotiation shaped by institutional discourses and professional values.

This article examines how identity tensions are shaped and sustained at micro, meso and macro levels, with a focus on the IP as a site of discursive struggle. In doing so, it highlights the broader sociocultural forces and power relations in academia and clinical practice, as well as the conditions under which IP is legitimised and reinforced. Doctoral education is a key pathway for developing expertise, yet phenomena such as impostorism can undermine confidence and belonging (Cawcutt et al. 2021; Clance and Imes 1978; Gill 2020; Tulshyan and Burey 2021; Wang and Li 2023). The purpose of this article is to reveal that IP is not an individual flaw, but a socially constructed effect of broader power relations, so that change can be understood and pursued at both individual and structural levels.

## 2 Background—Impostor Phenomenon and the Formation of Identity

First described by Clance and Imes (1978), IP reflects a persistent sense of intellectual fraudulence and a sense of achievement despite objectively determined achievement. Popularised as impostor syndrome, contemporary writings have returned to the term IP, signalling a recognition of the social, cultural, structural and political environments in which individuals exist. Indeed, the term impostor syndrome is considered misleading and suggests that this is a clinical condition (Bravata et al. 2020; Clance and Imes 1978; Coelho 2024; Dragon 2024). From a discursive perspective, IP can be understood as a socially and linguistically mediated phenomenon shaped by broader ideological structures.

Doctoral candidates commonly discontinue their studies due to inadequate supervision and mentorship, financial constraints (Devos et al. 2017; Rigler et al. 2017; Stirling et al. 2024), social isolation and lack of academic integration (Artiles and Matusovich 2020; Rigler et al. 2017; Wang and Li 2023), mental health challenges such as stress and depression (González‐Betancor and Dorta‐González 2020) and a mismatch between expectations and the realities of doctoral education (Devos et al. 2017). These challenges are common across doctoral education in multiple disciplines and are not unique to nursing. The process of identity formation provides doctoral candidates with a stronger sense of belonging, purpose and alignment with their academic community, which can mitigate feelings of isolation, reduce stress and help reconcile expectations with the realities of doctoral study (Devos et al. 2017; Henshaw et al. 2025; Lamichhane 2025; McAlpine et al. 2014; Mewburn et al. 2014). The cultivation of a scholarly identity can serve as a protective mechanism against these challenges, strengthening doctoral candidates' sense of purpose, confidence and perseverance (Devos et al. 2017; McAlpine et al. 2014; Owens and Godfrey 2022).

The term doctoral candidate is used in this paper to denote the specific phase of doctoral education most relevant to the experiences under investigation. Although empirical research seldom differentiates between doctoral students and candidates, the transition to candidature within the Australian context signifies a shift toward greater autonomy, heightened academic expectations, increased exposure to scholarly evaluation, potential reduction in supervisory and peer contact, and intensified identity negotiation (McAlpine et al. 2014; Mewburn et al. 2014).

### 2.1 Catalyst for Inquiry

Researcher positionality is central to doctoral studies, and its exploration is crucial for recognising how personal experiences and identities influence scholarly inquiry (Darwin Holmes 2020). Situated within a community of shared narratives, this work is informed by collective experiences, highlighting the role of relational knowledge building and the significance of supportive scholarly networks (Schalet et al. 2020). Motivated by challenges to their individual professional identity that emerged during the journey to and transition to doctoral candidacy, the first author sought to explore how identity is navigated in this space and how it is hindered by IP. As such, it became necessary to explore and critically interrogate how language creates spaces of impostorism for nurses, and how nurses may then reflect this back as a form of discursive legitimisation.

## 3 Methodology

Using Fairclough's critical discourse analysis (CDA) (Fairclough 1995, 2013), this study investigates how nursing doctoral candidates become positioned as ‘impostors’ through everyday discursive practices. This exploratory study aims to critically interrogate how language discursively constructs the IP for nurses undertaking doctoral study, challenging deficit‐based psychological framings by examining the sociocultural and ideological conditions through which impostor identities are legitimised and reproduced, and by creating space for more relational, reflexive and discursively informed educational practices.

Chosen for its framework, Fairclough's CDA allows for the examination of the relationship of power through language, which forms societal notions and concepts. That is, dominant discourses act to create social ‘truths’ where beliefs, norms, social positioning and ideologies become fact, rather than thought (Fairclough 2013). CDA is a socio‐political form of analysis that aims to uncover hidden forms of everyday power that marginalise population groups, thereby acting to emancipate them (Wodak and Meyer 2009). Indeed, CDA itself acts as an interrogation of power, influence and sociocultural and ideological structures that create meaning and truth. By investigating discursive formations, analysts engage in forms of resistance and advocacy, as forms of emancipation (Fairclough 1991, 2013). Fairclough's three‐dimensional framework (encompassing micro, meso and macro social orders) provides a unique platform upon which to investigate discursive truths and identify commonalities and divergences in the way discourse is described, consumed and enacted across individual, community and societal levels (Fairclough 1989, 1991, 1995, 2013).

### 3.1 Data Selection

Data from a range of sources were identified, ordered and synthesised. To identify data, a search was conducted in the Clinical Knowledge Network, CQUniversity Library, Google and Google Scholar using the prompts ‘professional identity’, ‘nursing doctorate’ and ‘impostor phenomenon’, as well as combinations and variations thereof. The final element of data was the use of reflective research memos, created by the first author in the journey to doctoral candidacy and as a doctoral candidate. After the data were identified, they were ordered according to their social order, as shown in Table 1. For example, magazines, news articles and first‐person accounts and personal reflections represented the micro level; articles from organisations and institutions, such as universities and professional bodies, represented the meso order, and theoretical literature represented the macro level. Data were allocated across micro, meso and macro social orders to make explicit the relational movement of impostor discourse between individual sense‐making, institutional mediation and broader ideological conditions, consistent with Fairclough's dialectical understanding of discourse (Fairclough 2013).

**Table 1:** Data across the social orders.

Social order	Order of discourse	Source of discourse/data	Dimensions of analysis
Micro	Discourse from doctoral candidates, particularly nurses	Personal reflections Magazine and news articles	Dimensions of meaning
Meso	Organisational discourse	Articles from universities and professional bodies	Structural effects
Macro	Theoretical literature	Published articles and eBooks	Broader social positioning

### 3.2 Analysis

**Figure 1:**
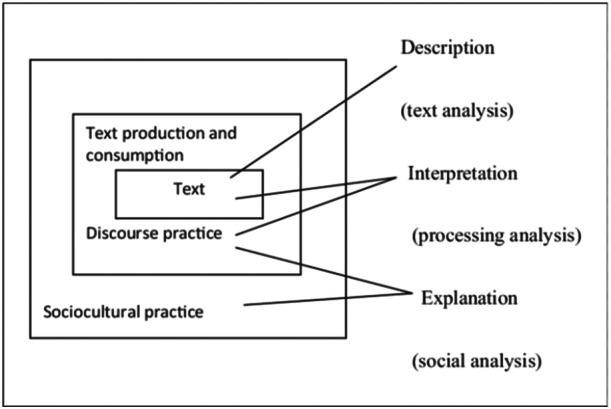
Fairclough's three‐dimensional framework. *Note:* Adapted from Fairclough's three‐dimensional model of critical discourse analysis (Fairclough 1995).

The analysis took a critical lens, focusing on IP and its wider relational positioning within the discourse. As shown in Figure 1, sources were analysed in relation to their dialectical relational properties (dimensions of meaning), features and structural effects (descriptive, interpretive and explanatory), as well as their broader social positioning (Fairclough 1989, 1991, 1995, 2013).

First, data were mapped in relation to the types of IP discourse and their underlying messages (see Supplementary Information S1). Data were considered in relation to their discourse type, description, discursive function and effect, ideology and social order. This iterative analysis enabled a focus on the sociocultural environment of professional identity and nursing, leading to the generation of the final topics, as demonstrated in Supplementary Information S1. This allowed for the interrogation of discursive positionings, which we have referred to as ‘themes’ for ease of reading.

## 4 Findings

The findings of this inquiry demonstrate that IP is often presented as an individual experience of those in deficit, rather than as a socially constructed experience. It is, however, disproportionately experienced by women, and as such, particularly impacts nursing candidates undertaking a doctoral program. Within nursing, historically shaped by gendered discourses of care and subservience, IP is reinforced by the longstanding devaluation of the profession and the persistent societal undervaluation of women's expertise. Supplementary Information S1 presents the data analysis, while Table 2 outlines the themes and subthemes derived from the data.

**Table 2:** Discursive themes and subthemes.

The impostor phenomenon as a socially constructed experience	Impostor phenomenon as a discursive construction of legitimacy and competence
	Impostor phenomenon as a gendered and discursively constructed experience
Impostor phenomenon as a symptom of wider system oppression	The legacy of care: Discursive constructions of nursing and reconstructing nursing identity
	Constructing professional identity: The impact of gendered expectations in nursing

### 4.1 The Impostor Phenomenon as a Socially Constructed Experience

#### 4.1.1 Impostor Phenomenon as a Discursive Construction of Legitimacy and Competence

The notion that IP is legitimised through language and positioned as an inherent attribute of the individual is highlighted by Gill (2020), for example (underlined for emphasis),

It is important that if you are afflicted by imposter syndrome, you should adopt effective techniques to help manage such feelings, so they do not adversely affect you…An important first step is to recognise that feeling like an imposter is largely related to your own perceptions of yourself.

(Gill 2020, 33)

Such a statement emphasises how IP is legitimised through language, framing it as an individual perception rather than a systematic concern. This sentence frames IP as an individual psychological issue, pathologising the experience, emphasising personal responsibility and personal deficit. Similarly, Zanchetta et al. (2020) frame IP as an individual psychological problem that necessitates self‐regulation and personal autonomy to be ‘overcome’ and to mitigate ‘negative outcomes’—‘This research suggests that interventions aimed at reducing the personnel consequences of the IP in the career and work context should focus on implementing a growth mindset’ (Zanchetta et al. 2020, 2). This perspective highlights the social positioning of IP as an attribute inherent to the individual, one that can be adjusted by cultivating a growth mindset, rather than as something that requires or addresses institutional change. It links the phenomenon to competence and self‐development (with an assumption that they start with a knowledge deficit), which are expected to be resolved through personal accountability.

The deficit of IP is evidenced in its continued comparison to competence.

Most doctoral students who have experienced IP indicate a lack of adequate academic preparation, specifically the ability of [*sic*] read, write, and academic think [*sic*]… Overall, the doctoral stage of study requires individuals to be able to cope with unknown challenges and ultimately complete their studies… In fact, many students may not understand the competencies requires [*sic*] for doctoral studies, how the educational process at the doctoral level works, or what it takes to successfully complete their studies… as far as the current education environment is concerned, a culture of genius seems to permeate all stages of learning.

(Wang and Li 2023, 5, 6)

Again, the discourse highlights how IP is framed and positioned as an inherent attribute of the individual, stemming from perceived deficits in preparation and competence. It reinforces the link between IP and the developmental stage of being a novice scholar, where struggle is framed as expected until sufficient expertise is gained. At the same time, the reference to a ‘culture of genius’ highlights how institutions contribute to these feelings, while the burden remains on the individual to cope. This discourse illustrates how institutional cultures legitimise such framing, which acts to reproduce and sustain these sentiments. As the first author observes [Author reflection], ‘I have heard many fellow PhD candidates who have been told “write more academically”, “use an academic voice”, “you need to find your academic voice”, and yet offering little insight into how this is developed’. Such interactions exemplify how institutional language positions candidates as lacking, reinforcing feelings of inadequacy and sustaining the discourse of IP.

The discourse of IP legitimises seniority by positioning novices as ‘naturally’ subject to impostor‐related beliefs and feelings until they achieve competence, thereby reinforcing hierarchical power relations. In doing so, it constructs seniors as competent, legitimate actors, while novices remain deficient and responsible for overcoming their perceived inadequacies. In discussing IP, Fowler and Villanueva (2023) reinforce the notion of legitimacy and position seniors as maintaining power, ‘nurses graduate from nursing schools clinically proficient but may lack leadership and team‐building skills. This may lead to an imbalance in the executive suite and result in nurse leaders developing imposter syndrome’ (Fowler and Villanueva 2023, e7). By tying impostor‐related beliefs and feelings to competence, specifically ‘leadership and team‐building skills’, it implies that nurses *should* experience IP until they acquire these skills, which reinforces the novice‐to‐senior progression. As Coelho (2024) observes,

Effective nurse leadership in healthcare requires the ability to delegate confidently. However, leaders experiencing imposter phenomenon can affect their team's productivity due to their difficulty in delegating and decision‐making… For example, with regards [*sic*] to task‐delegation decisions, leaders or managers who perceive themselves as frauds and have negative views of their own abilities can either over‐delegate tasks to avoid their perceived incompetence being exposed or under‐delegate tasks due to a lack of confidence in their own judgement.

(Coelho 2024, 29)

This perspective highlights how IP can also be framed as directly affecting not only the individual but also team performance, omitting the structural and cultural influences that often impact nursing leadership. By doing so, IP becomes a problem for the individual and a problem for those they lead.

Gill (2020) reported on IP for Early Career Nurse Researchers (ECNRs), stating,

not only is imposter syndrome relatively normal, especially for ECNRs, it arguably serves an essential purpose. An appropriate degree of self‐doubt and critical self‐reflection is integral to the research process ‐ it can improve quality and standards and reduce the likelihood of making significant errors… After all, only the arrogant have an unshakable view of their own value and influence…

(Gill 2020, 35)

This framing suggests that self‐doubt, as a form of deficit, is so deeply normalised within research culture that its absence becomes almost suspect, positioned as arrogance or overconfidence. In this way, the discourse not only reinforces impostor‐related beliefs and feelings but also constructs them as an expected, even desirable, attribute of early career development.

#### 4.1.2 Impostor Phenomenon as a Gendered and Discursively Constructed Experience

Since its initial conceptualisation, IP has been closely associated with high‐achieving women (Clance and Imes 1978). A recent meta‐analysis (Price et al. 2024) found that women consistently report higher levels of IP than men. Gender differences in IP have not decreased over time, despite shifts in gender stereotypes and women's representation in higher prestige fields, suggesting that these factors alone cannot fully explain the gap (Price et al. 2024). Critical analysis reveals that these differences are contextually constructed and mediated by field, geography and measurement approach, suggesting that the gendered experience of IP is shaped less by inherent traits and more by socially and discursively produced expectations. Thus, gendered patterns of IP should be interpreted in relation to their specific sociocultural context rather than assumed to be universal.

While the original conceptualisation of IP focused on individual cognition and personality traits, subsequent scholarship has broadened the lens, arguing that it reflects more complex and systemic factors (Wang and Li 2023), particularly gendered social norms. According to Wang and Li (2023),

Despite outstanding academic and professional accomplishments, women who experience the impostor phenomenon persist in believing that they are really not bright and have fooled anyone who thinks otherwise… and maintain low expectations for their own performance, as well as for other women… They have undoubtedly been instilled with family entrenched notions and self‐consolidated preconceptions of societal gender roles.

(Wang and Li 2023, 2)

While such a statement emphasises previously discussed individual deficit, it moves toward the emphasis of gender, framing it as both a personal and psychological affliction affecting women and reinforcing societal and familial expectations of gendered roles. The notion that IP is an inherent experience for women is highlighted by Clance and Imes (1978),

we do believe that the societal stereotype of women being less able intellectually than men begins to exacerbate and confirm at an early age the self‐doubts that have already begun to develop in the context of the family dynamics…if she were to acknowledge her intelligence, she would have to go against the views perpetuated by a whole society—an ominous venture indeed!

(Clance and Imes 1978, 243–244)

Here, IP is not simply a matter of professional identity but a reflection of the pervasive cultural scripts that position women as intellectually lesser across all spheres of life. The suggestion that acknowledging one's intelligence requires resisting ‘the views perpetuated by a whole society’ emphasises how deeply ingrained these stereotypes are in western cultural contexts, making the impostor experience a societal condition tied to the wider subordination of women rather than an individual or occupational issue. The reference to persevering ‘against tremendous odds’ reflects a power imbalance in which success requires resisting entrenched gendered subordination within these cultural contexts.

Evolving scholarship has challenged the early individualistic framing of IP, highlighting its prevalence among marginalised groups and its intersection with structural inequities, including sexism, racism, classism and xenophobia, and pointing to dynamics in western professional and academic contexts; however, heterogeneity in measurement and sampling means prevalence estimates vary, and research is growing globally (Bravata et al. 2020; Huecker et al. 2023; Tulshyan and Burey 2021; Wang and Li 2023). Tulshyan and Burey (2021) contend that dominant narratives about IP often obscure the sociohistorical contexts in which marginalised individuals navigate professional spaces, thereby reinforcing a discourse that individualises what are, in fact, systemic forms of exclusion. When viewed through the lens of gendered and societal stereotypes, IP is often framed as an intrinsically female affliction, both a personal and psychological burden tied to women, particularly in western cultural contexts. This framing is especially relevant to nursing, a female‐dominated profession in many western contexts, where such dynamics of power and subordination position nurses as subordinate to men, and leave even doctorally prepared nurses vulnerable to impostor‐related beliefs and feelings.

The notion of the subordination of women nurses is highlighted by Evans (1997) and reinforces how patriarchal structures in western cultural contexts disadvantage women, framing career progression as constrained by gendered expectations. It demonstrates how power and subordination, particularly in nursing, where women are burdened with gendered stereotypes, while men are considered free to advance professionally. For example,

men nurses distancing themselves from women colleagues and allying themselves with men physicians, patriarchal gender relations that value masculine character and leadership traits are perpetuated and maintained… This is accomplished by devaluing the feminine, and consequently poses a significant barrier to women's attainment of leadership positions.

(Evans 1997, 229)

This aligns with the argument that patriarchal structures devalue femininity and privilege masculine traits, particularly in western professional contexts. In nursing, while women hold leadership positions, they may adopt masculine behaviours and leadership styles that are valorised within institutional and organisational practices, reflecting the need to conform to entrenched norms of authority (Pincha Baduge et al. 2024).

### 4.2 Identity Formation in the Nursing Doctoral Candidate: The Impostor Phenomenon as a Symptom of Wider System Oppression

While this inquiry aimed to investigate the framing of IP in relation to nursing doctoral candidates, the discourse revealed broader influences that discursively frame nurses (as a profession, as women and as doctoral candidates) in western cultural contexts. Given the social framing of IP presented above, understanding how it impacts the identity and success of the nursing doctoral candidate is particularly pertinent.

#### 4.2.1 The Legacy of Care: Discursive Constructions of Nursing and Reconstructing Nursing Identity

Due to the language that continues to frame the nursing profession, nursing doctoral candidates are likely to experience IP at a striking rate, as their work is continuously filtered through discourses that emphasise care and service. The traditional associations of nursing as ‘carers’ and ‘mothers’ reinforce these expectations, drawing on the profession's roots in domesticity and moral duty (Farr 1990). While men have increasingly entered nursing, the profession remains discursively constructed as women's work, tied to the ‘soft skill’ of care, relational competence, human‐centred skills, compassion and emotional labour (Evans 1997; G. Fealy et al. 2013; G. M. Fealy 2004; Nelson and Rafferty 2011; Nyborg and Hvalvik 2022). This framing not only shapes how nurses are perceived but also influences how they experience more masculinised domains such as academia, where authority, rationality and intellectual autonomy are privileged (Australian Nursing and Midwifery Journal 2024; van Dongen and Hafsteinsdóttir 2022; Fowler and Villanueva 2023; Pincha Baduge et al. 2024). Thus, nursing doctoral candidates are repeatedly reminded by the discourse of their ‘natural feminine attribute’ to ‘care’, even as they attempt to assert themselves in academic and leadership spaces (Clayton‐Hathway et al. 2020, 62).

Nursing has historically been constructed through gendered and sociocultural discourses that emphasise care, morality and service (Farr 1990). Shaped by nineteenth‐century ideals of womanhood and social duty, early nursing models promoted obedience and altruism over intellectual autonomy, reinforcing the profession's subordination within patriarchal and biomedical hierarchies (Evans 1997; G. Fealy et al. 2013; G. M. Fealy 2004; Nelson and Rafferty 2011; Nyborg and Hvalvik 2022). These narratives have obscured the knowledge‐based and political dimensions of nursing, framing it as a vocation rather than a site of knowledge production (Traynor 2017). As a result, nurses, particularly those entering academic or leadership roles, may experience impostor‐related beliefs and feelings as they navigate professional spaces that have historically devalued their authority (Australian Nursing and Midwifery Journal 2024; Doleman et al. 2024; van Dongen and Hafsteinsdóttir 2022; Fowler and Villanueva 2023; Pincha Baduge et al. 2024). Evans (1997) argues that,

of great significance is the role played by Florence in the 19th century in firmly establishing nursing as a female occupation. To Florence Nightingale, every woman was a nurse, and women who entered nurses' training were doing only what came naturally to them as women… The apprenticeship style of education she subsequently initiated for nurses was based on these beliefs, for it was deemed that women did not require education prior to working in hospitals. Instead, they would learn on the job under the tutelage of men physicians. The belief that nursing was an extension of women's domestic roles was consequently instrumental in establishing nursing as not only a woman's occupation, but as one that was unskilled and of low value in comparison to male occupations, particularly medicine.

(Evans 1997, 228)

This quote illustrates how, in a western patriarchal cultural context, the historical framing of nursing as a ‘natural’ extension of women's roles as caregivers and mothers sets the stage for or legitimises the conditions for nursing doctoral candidates to experience IP at an intensified rate.

For doctoral nurses, identifying with femininity and care creates identity dissonance. This identity dissonance is where professional advancement conflicts with entrenched gendered expectations. Allan et al. (2008) remind us that ‘nurses feel themselves devalued socially, and that, globally, nursing is not given the same status as other, socially more prestigious professions, such as medicine’ (Allan et al. 2008, 555), a view particularly relevant when considering identity formation and female‐nursing subordination in historically masculine domains. By emphasising this, Allan et al. (2008) highlight how nursing is discursively devalued in comparison to other professions, reinforcing its roots in feminised traditions of care and positioning it as socially subordinate. For doctoral nursing candidates, such language intensifies the IP by continually reminding them of nursing's association with maternal, relational acumen, caregiving, rather than the more prestigious ‘masculine’ domain of academia. As the first author observes, [Author reflection] ‘Why are you doing a PhD when you have a family?’ Because nursing has historically been associated with women, nursing doctoral candidates are often discursively positioned as women first and scholars second. In this way, IP can be understood within a gendered order. Discourse may reinforce impostor‐related beliefs and feelings by privileging ‘feminine’ obligations alongside professional identities, though the degree of this effect will vary across contexts and individuals.

According to Allan et al. (2008), ‘the lack of adequate recognition of nursing as a profession has led to many people regarding nursing as a too inferior and inadequate undertaking to be regarded as a “profession”’ (Allan et al. 2008, 550). By framing nursing as inferior and inadequate, the quote exposes how gendered assumptions devalue both the discipline and its practitioners and, thus, its researchers, ensuring they are continually reminded of their socially inscribed role as carers rather than scholars. According to Gill (2020), ‘the fact the nursing discipline remains a relative research neophyte perhaps further compound [*sic*] potential feelings of uncertainty’ (Gill 2020, 33). ‘Research neophyte’ is an interesting term and language that compounds IP for doctoral candidates, as the discipline's academic legitimacy is discursively undermined. The term *‘neophyte’ combines ‘neo’ (new) and ‘phyte’ (plant), suggesting something newly planted or immature, which frames nursing as still developing or underdeveloped* in its research identity. Rooted in the tradition of nursing as a feminised, care‐based role, this language reinforces doctoral candidates' impostor‐related beliefs and feelings. At all times, they are reminded of their socially inscribed identity as carers rather than scholars, amplifying their vulnerability to IP.

Resistance to this, however, is an option. According to Allan et al. (2008),

for the value of nursing to be recognized in terms of national and international policies regarding future health and social care, nursing as a profession needs to see value in its work and make this a driving force; that is, the value of nursing itself is in building and becoming a realistic and successful profession with a voice in governments and on the world stage, putting nursing on the map.

(Allan et al. 2008, 554)

This perspective argues that nursing must actively resist its traditional framing as a feminised, caring role by asserting its professional value and authority in policy and academic spaces. Until nursing claims this recognition, doctoral candidates remain vulnerable to IP as they navigate tensions between the expected ‘feminine’ care identities and the pursuit of scholarly legitimacy.

#### 4.2.2 Not a ‘Real’ Doctor; the Many Locations of the Doctoral Nurse

As highlighted above, nurses are firmly positioned as caring females within healthcare, and when they move into academia, they are re‐subordinated within its hierarchy, thereby being reframed as impostors. The use of the title ‘Doctor’ within healthcare is more than just a label; it is deeply embedded in historical, institutional and ideological practices. Tracing its etymology to the Latin *‘docere’*, meaning *‘to teach’*, the term was initially reserved for scholars in law and extended to teachers of medicine, theology and philosophy in the thirteenth century (Asfour and Winter 2018; Lawrence 2011). Over time, particularly during the professionalisation of medicine in the eighteenth and nineteenth centuries, the title ‘Doctor’ became increasingly standardised within medical schools (Lawrence 2011). This historical development continues to shape contemporary understandings of who may legitimately claim the title. As such, nurses moving into doctoral space are faced with discourses surrounding the use of the title, a subtle way in which power is enacted, particularly as it is linked to the scope of practice in a health setting, and nurses are othered.

In modern healthcare discourse, the title Doctor remains a contested terrain, particularly in clinical settings where it is strongly associated with medicine. While individuals in other health professions, such as nursing, may hold doctoral qualifications, their entitlement to use the title is often questioned. Institutional policies, cultural expectations and historical precedents reinforce the idea that only medical doctors should assume the designation to avoid patient confusion or conflict with professional traditions (Asfour and Winter 2018; Blake 2023; Collier 2016; Edmonds et al. 2023; Taylor 2023). For example, Gaddis (2022) asserts that ‘so that confusion in clinical settings regarding just what “doctor” means can be avoided in the future’ (Gaddis 2022, 320). Such a statement highlights how, despite holding a doctorate, nurses are discursively subordinated, and consumers of care are assumed to be incapable of understanding nuances in titles. The notion and discourse that a nursing doctorate is an impostor qualification is highlighted by Gardenier (2011) and reinforces delegitimisation of nursing doctorates: ‘patients rely on health care providers' professional designations as an indication of the level of training, skills, and knowledge of those providing their care. The use of the prefix “Dr” or “Doctor” by NPs who have completed the DNP degree could lead to confusion and misconceptions by patients’ (Gardenier 2011, 562). This perspective illustrates how, despite holding doctoral qualifications, nurses are discursively denied complete legitimacy, as their use of the title is framed as misleading or confusing. A documented misconception is that nurses who pursue doctoral‐level studies do so to impersonate physicians in the clinical setting and do so fraudulently (Gaddis 2022). Gaddis (2022) asserts the notion of fraudulence in nurses who achieve doctoral qualifications ‘The fraud exists because in claiming the title “Doctor” in a clinical setting, the DNP who is in essence impersonating a physician ignores the substantial knowledge and training gaps that exist between a physician and a non‐physician’ (Gaddis 2022, 314). Such positioning entrenches their subordination within the healthcare hierarchies, where even the most qualified nurse is denied parity with medical doctors, reinforcing the sense that nursing doctorates can never confer equivalent status and recognition. This is not a claim of equivalence in the scope of practice; medical qualifications are professional clinical degrees, followed by years of postgraduate clinical training including internship, supervised practice, specialty training and ongoing credentialling requirements, whereas doctoral qualifications in nursing are research or advanced practice‐focused degrees emphasising scholarship, evidence‐based practice and leadership (Halabicky et al. 2024; Negarandeh and Khoshkesht 2022; Waldrop et al. 2023). Despite these different aims and training pathways, institutional and public norms in clinical settings tend to associate the title ‘Doctor’ primarily with medical doctors, thereby contributing to the discursive delegitimisation of doctorally prepared nurses.

Such claims warrant critical interrogation. While the use of the title ‘Doctor’ is often justified by appeals to tradition, this masks a modern conflation of the title ‘Doctor’ as synonymous with clinical authority, when in fact the original association meant a scholar or one who teaches, and in doing so, it privileges medical status while sidelining other health professionals (Asfour and Winter 2018). This discursive struggle is visible not only in everyday clinical interactions but also in the academic literature, where arguments over the appropriateness of the title expose deeper tensions concerning professional boundaries, authority and legitimacy. These arguments often reflect deeper issues around professional identity and power. Medicine has long held the dominant voice in healthcare, and restricting the use of the title ‘doctor’ can reinforce this dominance (Bowker‐Howell et al. 2024).

Doctorally qualified nurses occupy a distinct epistemic space defined by advanced research, leadership and specialised knowledge (van Dongen and Hafsteinsdóttir 2022; Gaddis 2022; Waldrop et al. 2023; Walker et al. 2016). Yet restrictions on using the title ‘Doctor’ reproduce professional boundaries and asymmetrical power that privilege medicine, undermining nurses' academic and clinical achievements and fostering feelings of illegitimacy and impostorism despite their demonstrated competence.

Impostor phenomenon can be linked to structural and cultural forces that question one's legitimacy or belonging, particularly in environments marked by entrenched hierarchies (Bravata et al. 2020; Clance and Imes 1978). As such, the denial of title usage is not a neutral or purely semantic issue; rather, it reflects broader power imbalances that can profoundly affect how nurses perceive their role, authority and worth within interdisciplinary healthcare teams.

## 5 Discussion—Identity Formation and Impostor Phenomenon

This article aimed to examine the construction of nurse‐academic identity within the context of IP. From a discursive perspective, it is best understood as a socially and linguistically mediated phenomenon shaped by wider ideological structures. While the analysis of this article focused on the discursive positioning of IP from a top‐down framing, discourse is a cyclic process whereby language makes people and people make language. As such, doctoral nurses often reflect such framing back as they navigate the social positioning within their educational and professional lives. Indeed, candidates may frequently reflect dominant academic and professional discourses back through their language, practices and self‐positioning, often normalising impostor experiences as expected, inevitable or even desirable markers of scholarly development (Fairclough 1989, 1991, 1995). By internalising narratives that frame self‐doubt as a sign of humility, competence‐in‐formation or intellectual rigour, candidates may come to narrate their experiences in ways that align with institutional expectations, thereby reinforcing the legitimacy of these discourses. This reflection operates as a form of discursive compliance, in which candidates reproduce deficit‐oriented framings through everyday academic interactions, such as supervision discussions, progress reporting and peer conversations, even when these framings undermine their sense of scholarly authority. In this way, the IP is not only imposed through institutional or disciplinary discourse but also sustained through its iterative uptake and rearticulation by nursing doctoral candidates themselves. Importantly, this process does not negate agency; rather (as the analysis suggested), it highlights how agency is exercised within constrained discursive possibilities, where reproducing dominant narratives may function as a strategy for belonging, legitimacy and survival within doctoral and academic spaces.

The analysis suggested that IP is commonly described as an individual deficit. Referring to the phenomenon as predominantly individual fails to recognise that these perceptions do not emerge in a vacuum; broader structural, cultural and relational dynamics, including systemic inequality, professional socialisation processes and unrealistic standards, shape them (Clance and Imes 1978; Jang et al. 2025; Parkman 2016). Through a critical analytical lens, the findings reveal a discursive tension between prevailing professional expectations and the creation and maintenance of the identity of a doctoral‐prepared nurse and how that identity is formed. The discourse of IP reproduces existing power structures by individualising systemic issues and shifting the accountability and responsibility away from the system and toward the individual (Fairclough 1995, 2013).

Nurses experience persistent subordination in healthcare hierarchies, evident in limited political and organisational leadership, restricted decision‐making participation and exclusion from socio‐political arenas, reflecting discursive marginalisation (Doleman et al. 2024; Gunn et al. 2019; Stirling et al. 2024). These dynamics are reinforced by medical (and other professional) dominance, internalised nursing standards and gendered discourses that frame nursing as ‘women's work’, undervaluing expertise despite high skills and education, while historical ideals of virtue, morality and self‐sacrifice continue to shape professional identity (Allan et al. 2008; Kathleen Croft and Anne Cash 2012; Evans 1997; Nelson and Rafferty 2011; Nyborg and Hvalvik 2022; Pincha Baduge et al. 2024). Doctoral education offers nurses a chance to assert intellectual authority and reclaim professional legitimacy, yet the intersection of nursing, gender and academia fosters IP, undermining scholarly identity.

The recurring discourse of self‐doubt and perceived fraudulence aligns with societal expectations of exceptional performance and infallibility, often rooted in gender and class. For nurses, this contributes to professional marginalisation and challenges the development of a confident academic identity. Limited institutional support, underfunding of nursing research and discouragement from peers regarding doctoral study exacerbate these dynamics, prompting doctoral candidates to question the value of their contributions and their place within academia (Henshaw et al. 2025; Mourão Netto 2023; Pincha Baduge et al. 2024). Gendered norms intersect with professional expectations, producing a discursive tension between individual aspiration and structural constraints. Women's dominance in nursing does not translate into leadership parity; men occupy a disproportionate number of senior roles, and cultural assumptions about femininity influence evaluations of competence (Kathleen Croft and Anne Cash 2012; Doleman et al. 2024; Hughes and Clancy 2009).

These systemic and relational barriers have consequences that extend beyond the individual. Restricting the recognition and authority of doctorally prepared nurses limits the profession's capacity for research leadership, the development of evidence‐based policies and influence on healthcare outcomes, ultimately constraining nursing's evolution and societal impact (Halabicky et al. 2024). Recognising this, doctoral education and professional advocacy can become transformative tools: by increasing visibility in research, leadership and innovation, nurses can assert authority, reclaim knowledge production and challenge the discourses that foster impostor‐related beliefs, feelings and experiences (Dobrowolska et al. 2021; Gardenier 2011; Henshaw et al. 2025). In doing so, doctorally prepared nurses not only advocate for their professional identity but also shape the frameworks through which healthcare practices are understood and delivered, highlighting the critical link between individual empowerment and systemic change.

Doctoral nurses challenge traditional hierarchies by asserting evidence‐informed perspectives grounded in frontline experience and expanding the nursing voice through mentorship and education (Negarandeh and Khoshkesht 2022). These contributions lead to more equitable, holistic and context‐sensitive care, redefining the professional identity and influence of nursing within the healthcare system (Dobrowolska et al. 2021; Gill 2020; Henshaw et al. 2025). Despite these challenges, doctoral education and professional advocacy offer pathways to disrupt these discourses. Indeed, the discourse of impostorism requires considered reflection: what do we mean when we say IPs? Does the label ‘impostor phenomenon’ create a discursive relationship that subordinates women or nurses reaching for greater heights? Can the discourse be reframed as a learning opportunity, rather than a deficit? By interrogating and challenging narratives that individualise failure and devalue nursing expertise, nurses, candidates, supervisors and institutions can reconfigure the positionality conundrum for nurses and the IP within both clinical and academic spheres. Increased visibility in research, leadership and innovation signals potential for systemic change, empowering doctoral nurses to assert authority, reclaim knowledge production and mitigate impostor experiences (Dobrowolska et al. 2021; Gardenier 2011; Henshaw et al. 2025). Ultimately, doctoral education becomes a vehicle not only for professional advancement but for structural transformation, enhancing nursing's influence and improving patient care outcomes (Australian Government 2019; Dobrowolska et al. 2021; van Dongen and Hafsteinsdóttir 2022; Henshaw et al. 2025; Lamichhane 2025; Negarandeh and Khoshkesht 2022; Stirling et al. 2024; Waldrop et al. 2023). The discourse of impostorism has created a social chasm within which many can be lost. Language, support and action against this discourse can bridge this gap.

Taken together, these insights invite a fundamental reconsideration of how the IP is understood within doctoral education and nursing more broadly. Rather than interpreting impostor experiences as evidence of personal deficit or psychological vulnerability, this analysis suggests they may reflect a common, if uncomfortable, feature of educational transition, identity negotiation and learning at the boundaries of knowledge. Crucially, however, recognising uncertainty, difficulty or developmental struggle need not require the discursive production of illegitimacy. When doctoral nurses are encouraged, implicitly or explicitly, to interpret uncertainty as imposture, self‐doubt becomes moralised, and belonging is rendered conditional. This paper argues, therefore, that the problem is not that doctoral nurses experience moments of doubt, but that academic and professional discourses render such moments intelligible only through the language of fraudulence and deficiency. Reframing the IP as a socially produced effect of discourse creates space to acknowledge uncertainty as an ordinary component of scholarly formation, without positioning nurses as less legitimate, less capable or perpetually becoming. Such a shift has important implications for how doctoral education is conceptualised, supported and narrated, challenging institutions and supervisors to disrupt discourses that normalise self‐doubt while simultaneously undermining the legitimacy of those who experience it. Indeed, reframing imposture as belonging underscores the identity shifts required in doctoral becoming and opens space for candidates to reinterpret self‐doubt as a meaningful part of the process.

Nurses and doctoral nursing candidates inevitably perceive the IP in diverse ways, shaped by their individual experiences, contexts and professional development stages. This variability underscores that there is no singular or fixed interpretation of the construct. While the IP originally offered insight into experiences of self‐doubt and perceived inadequacy, it may inadvertently function as a limiting framework that reframes imposture against belonging. Such framing risks constraining identity. However, this notion can be disrupted through critical reflection of all involved in such a framing. Nurses and doctoral candidates can move beyond deficit‐based interpretations, instead extending and reconfiguring existing knowledge and professional practice in ways that affirm capability, agency and scholarly contribution. In doing so, identity may be better formed and supported, fostering a sense of belonging and learning.

## 6 Limitations

The study included personal reflections by the first author as part of the data, which were extracted as excerpts from critical reflections and reflective memos. We acknowledge this is a tension between CDA and autoethnographic practice. While these reflections provided valuable insight into the research context, their inclusion introduces a methodological tension. CDA traditionally emphasises rigorous, systematic examination of communicative messages, whereas the use of personal reflections aligns more closely with autoethnographic approaches, which carry specific methodological requirements to ensure credibility and mitigate bias. Acknowledging this methodological complexity is important because it highlights the need for careful reflexivity and transparency in generating and using personal reflections as data.

## 7 Conclusion

This discursive piece demonstrates that the IP among nursing doctoral candidates is not an individual psychological deficit, but a socially and discursively constructed experience. By tracing how language, power and institutional norms shape candidates' identities, particularly around gender, caring and knowledge ownership, it highlights the structural mechanisms that delegitimise nurses' participation in higher education. It is therefore imperative that nursing doctoral candidates critically interrogate and transcend these internalised barriers throughout the doctoral trajectory. This process necessitates sustained engagement in critical reflection, systemic synthesis of existing evidence, rigorous evaluation and the strategic development, adaptation and implementation of research methodologies aimed at extending and reconfiguring extant knowledge and professional practice. Reconceptualising impostor experiences through a critical discourse lens foregrounds the need for institutions to actively transform the cultures, policies and practices that reproduce these inequities.

Integrating opportunities for critical reflexivity into doctoral education may enable candidates to examine and reshape the discourses that shape academic identity formation, thereby supporting the development of confident and empowered nurse scholars. Finally, future research should explore concrete discourse‐based interventions capable of reshaping doctoral education and dismantling the social production of impostor subjectivities.

## Author Contributions

**Chantel Sando:** conceptualisation, methodology, analysis, investigation, writing – original draft, project administration. **Amy‐Louise Byrne:** supervision, methodology, analysis, validation, writing – review and editing. **Adele Baldwin:** supervision, conceptualisation, writing – review and editing. **Pauline Calleja:** supervision, writing – review and editing.

## Ethics Statement

The authors have nothing to report.

## Conflicts of Interest

The authors declare no conflicts of interest.

## AI Declaration

The authors declare that no generative artificial intelligence (AI) tools were used to generate content for this manuscript.

## Supporting information


Supporting File

## Data Availability

The data that support the findings of this study are available from the corresponding author upon reasonable request.
