# Supplementary material for: Constructing the Impostor and Navigating Identity: A Critical Discursive Exploration of the Impostor Phenomenon and Nursing Doctoral Candidates
Source: Nurs Inq. 2026 Aug 2;33(4):e70144. doi: 10.1111/nin.70144 (PMC13429040; doi:10.1111/nin.70144)
Supplement: Supplementary file 1 — Supporting File [file NIN-33-e70144-s001.docx]

**Supplementary Information 1**

Initial analysis and grouping of themes

| **Discourse Type** | **Description** | **Discursive Function or Effect on Subjectivity** | **Implied Ideology or Underlying Assumption** | **Social Order/s** | **Examples** |
| --- | --- | --- | --- | --- | --- |
| **Impostor phenomenon as a psychological experience** | Positions impostor phenomenon as an individual problem to be solved through personal change. | Individualises responsibility, depoliticises systemic and structural barriers, silences collective struggle. | Success and confidence are matters of mindset; structural challenges are irrelevant. | Dominantly, the Micro level. The focus is on the individual experience | *“It’s best to try and manage imposter syndrome as it can hinder you from pursuing new opportunities, including mentoring students, involvement in union activities and career progression” (Dragon, 2024, p. para.4)*  *“there is robust literature that describes the harmful association between impostor feelings and job performance, job satisfaction, and burnout among various employee populations, including healthcare professionals. In light of this evidence, we encourage professors and employers to incorporate recognition of this phenomenon in the development of both structured (e.g., training, orientation, onboarding) and unstructured (e.g., mentoring, coaching, self-directed learning) learning and career development activities. Success-oriented employees such as clinicians commonly have a thirst for training and personal growth. Offering resources such as access to therapy and resilience trainings [sic] that focus on impostor syndrome could help reduce the prevalence of impostorism in employed populations.” (Bravata et al., 2020, p. 1272)* |
| **Legitimisation of the impostor in academic identity formation** | Frames impostorism as a routine stage of academic development. | Minimises structural barriers, dismisses the legitimacy of self-doubt, and imposes an implicit standard of assumed competence. | Self-doubt is natural and expected; humility safeguards against mistakes.  (1) Self-doubt is inevitable and natural  (2) Competence should already be assumed. | Micro and Meso levels. It relates to the departmental and academic environment, shaping identity. | *“The impostor phenomenon is common among doctoral students” (Wang & Li, 2023, p. 02)*  *“The academic environment is highly competitive, which invariably results in many researchers comparing themselves with others and concluding their colleagues are more capable and intelligent than they are” (Gill, 2020, p. 32).*  *“Imposter syndrome appears to be ubiquitous in academia” (Gill, 2020, p. 34)*  *“The process of transition from a learner to a researcher is akin to a rite of passage, requiring a liminal period of uncertainty, confusion, or doubt to achieve a change in identity” (Wang & Li, 2023, p. 05)*  *“It is important to recognise that not only is imposter syndrome relatively normal, it is an indispensable component of scholarly activity that can ground and motivate us when managed appropriately” (Gill, 2020, p. 35).*  *“Impostor Phenomenon is alive and well on college campuses. While more empirical research is necessary to fully understand IP, particularly with regard to faculty and staff, there is enough documentation to support the integration of programming on campus and reflection upon how the academy feeds it” (Parkman, 2016, p. 56)*  *“It is crucial to note that Impostor Syndrome stems not just from the mismatch between the representation of an academic and one’s identity, but also from the daily experiences in which faculty, students, and administrators convey that you don’t belong, or that you don’t have what it takes.” David Leonard cited in Parkman (2016, p. 57).*  *“In some cases, impostor phenomenon provides motivation for success” (Jang et al., 2025, p. 7)* |
| **Perfectionism, professionalism, and/or meritocracy** | Constructs academic success as requiring flawless performance, composure, and constant competence. | Creates perpetual pressure to perform, naturalises fear of failure, and obscures systemic inequities. Discourages openness to failure. | Only perfection and resilience earn legitimacy; errors are personal failings. | Dominantly, the Macro level. It reflects both structural and disciplinary expectations, as well as societal norms. | *“Hutchins noted that those attracted to higher education, and the current work environment in these settings, align closely to the factors that contribute to the development of impostor tendencies. IP is more common in those with traits of conscientiousness, achievement orientation, perfectionistic expectations, and those working in stressful and highly competitive professions” (Cawcutt et al., 2021, p. 645)*  *“Critical feedback and rejection are common. You often have to apply multiple times to get published, funded or employed, so it is prudent to get used to the cycle of production, rejection, revision and acceptance, as it is completely normal. Peer review is a natural part of academic life. While it may occasionally be destructive, it is usually helpful in developing work further. It is also here to stay. It is therefore important to learn how to deal with criticism, however harsh it may be”(Gill, 2020, p. 34).*  *“IP is more often seen in those with advanced degrees, those who have the traits of conscientiousness, achievement orientation, perfectionistic expectations, and people who work in highly competitive and stressful occupations” (Parkman, 2016, p. 53)* |
| **Stoicism or masking** | Encourages concealment of doubt and emotional restraint. | Individualises responsibility, suppresses vulnerability, silences open discussion of struggle. | Doubt must be hidden; success requires composure and masking. | Micro and Meso levels. Focuses on individual coping strategies and concealment of emotions. | *“The client needs to become aware of the*  *superstitious, magical aspects of her impostor*  *belief and must consciously experiment with*  *changing her ritualistic behaviors. For example,*  *she is encouraged to study for an exam with the*  *expectation, "I will do well on this exam"*  *rather than, "I may fail." When she is able to*  *succeed without the self-doubting beforehand,*  *she has made a major breakthrough in undoing*  *her ritual of predicting failure”(Clance et al., 1978, p. 245).*  *“If IS has been identified as a barrier, reframing and cultivating positive micro-affirmations can potentially foster individual growth and confidence. Dissolving deeply rooted feelings of inadequacy requires targeted actions that increase self-confidence. These actions, called “reframing,” can include actions such as appreciative inquiry, recognition, validation of experiences/ feelings, reinforcing/rewarding positive actions, requesting inclusion in important professional meetings, and designing a “hero” wall in the workplace with certificates, photos, and accomplishments” (Fowler & Villanueva, 2023, p. e9)*  *After all, success is public but failures are largely private, so the only failures we ever really see are our own. Consequently, when we observe other researchers justifiably celebrating their successes, it is usually just the tip of the proverbial iceberg…we rarely see their struggles and failures, despite such challenges constituting much of academic life for even the most successful researchers.” (Gill, 2020, p. 34).* |
| **Exceptionality discourse** | Positions academic success as the domain of the extraordinary few. | Excludes “ordinary” striving, fostering elitism, and reproduces hierarchies of worth. | Only the most intelligent and exceptional belong in scholarly roles. | Dominantly, the Macro level. Reflects structural hierarchy and societal valuation of ‘exceptional’ scholars. | *“Most people who are experts in their field are usually experts because they never stop learning”(Gill, 2020, p. 34)*  *“In fact, many students may not understand the competencies requires [sic] for doctoral studies, how the educational process at the doctoral level works, or what it takes to successfully complete their studies. “Drifting” into doctoral education can exacerbate feelings of unease, incompetence, and low self-esteem. In addition, doctoral students generally face multiple dilemmas such as difficult coursework, an increasingly competitive academic environment, and low levels of social support, which contribute to a challenging and stressful doctoral phase” (Wang & Li, 2023, p. 05)*  *“Max Weber regarded the academic profession at that time as a “calling”… highlighting the sacred status of academic work and its value implications. Currently, many Ph.D. students enter the academic world with enthusiasm but cannot break free from the continuous evaluation, monitoring of results, and glorification of excellence, some doctoral students might begin to demand too much from themselves and feel like an impostor who is not good enough for doctoral studies” (Wang & Li, 2023, p. 06)* |
| **Gendered caring versus gendered scholarship** | Positions nursing identity as caring practice, delegitimising academic and leadership pursuits. | Undermines scholarly ambition, narrows definitions of “real nursing.” | Nursing is inherently about bedside care; academia, however, is often detached from reality. | Micro, Meso and Macro levels. It links professional practices, departmental and organisational, to broader societal gender norms. | *“The concept of ‘women’s work’ is based on essentialist notions of gender which prescribe that women and men have different characteristics which are inherently biological. This belief has profoundly shaped the way work is valued in our society and, because of its association with ‘natural’ feminine attributes such as love, care and empathy, nursing is viewed as an occupation particularly suitable for women… Assumptions that caring is a natural feminine trait have long influenced the development of nursing as a profession, and the level and type of training and education required” (Clayton-Hathway et al., 2020, p. 19).*  *“All nurses have personal reasons for being nurses, with the common aim and value being caring for individuals, but also for humanity at large” (Allan et al., 2008, p. 555).*  *“The gendered construction of nursing leaves a legacy which continues to feed the current crisis, including suppressing wages and downgrading working conditions. Historic perceptions that care is a naturally feminine skill or characteristic sit in direct opposition to the high level of skills and professionalisation required in contemporary nursing” (Clayton-Hathway et al., 2020, p. 5)* |
| **Gendered disciplinary or gendered professional identity in nursing** | Constrains ambition and intellectual authority, especially for women | Reinforces patriarchal norms and limits professional advancement. | Women should remain modest; ambition is often seen as a contradiction to femininity. | Dominantly, the Macro level. Focuses on societal patriarchy and structural gender norms. | *“in female-dominated occupations such as nursing, patriarchal gender relations which reﬂect a high valuation of all that is male and masculine, play a signiﬁcant role in situating a disproportionate number of men in administrative and elite specialty positions. At the heart of this gender dynamic is the need to separate the masculine from the lesser valued feminine. Male nurses do this by employing strategies that allow them to distance themselves from female colleagues and the quintessential feminine image of nursing itself, as a prerequisite to elevating their own prestige and power. They are aided in this task by patriarchal cultural institutions that create and perpetuate male advantage, as well as by women nurses themselves who, consciously or unconsciously, nurture the careers of men colleagues”(Evans, 1997, p. 226).*  *“Gender discrimination exists in nursing, with men having a higher rate of pay and a faster rate of career progression, irrespective of experience and qualifications. In addition, men are shown to have a greater representation in leadership roles compared to the proportion of men in the profession” (Doleman et al., 2024, p. 375).*  *“Rising in their careers faster than women co-workers was attributed to informal men networks, societal gendered expectations, and a men-dominated system under scoring ongoing patriarchy and sexism rather than considering merit. Some men nurses acknowledged their gender gave them greater opportunities for career advancement. When women nurses did aspire to leadership positions, they reported having to work harder to prove themselves, felt undervalued and invisible in their workplaces, and were held to higher standards than their men counterparts” (Pincha Baduge et al., 2024, p. 9).* |
